# Supplementary material for: Deep mutational scanning and machine learning reveal structural and molecular rules governing allosteric hotspots in homologous proteins
Source: eLife. 2022 Oct 13;11:e79932. doi: 10.7554/eLife.79932 (PMC9662819; doi:10.7554/eLife.79932)
Supplement: Supplementary file 1. [file elife-79932-supp1.docx]

Supplementary File 1. Pairwise sequence identity and similarity

| Protein pair | Identity | Similarity |
| --- | --- | --- |
| TetR vs. MphR | 14.8% | 26.8% |
| TetR vs. RolR | 16.7% | 22.5% |
| TetR vs. TtgR | 19.2% | 30.4% |
| MphR vs. RolR | 17.1% | 29.7% |
| MphR vs. TtgR | 17.7% | 36.7% |
| RolR vs. TtgR | 17.6% | 29.8% |
