# Supplementary material for: Deep mutational scanning and machine learning reveal structural and molecular rules governing allosteric hotspots in homologous proteins
Source: eLife. 2022 Oct 13;11:e79932. doi: 10.7554/eLife.79932 (PMC9662819; doi:10.7554/eLife.79932)
Supplement: Supplementary file 2. [file elife-79932-supp2.docx]

Supplementary File 2. R-squared correlation of deads identified at each position between replicates.

|  | **Replicates** | | |
| --- | --- | --- | --- |
|  | R1 / R2 | R1 / R3 | R2 / R3 |
| TetR | 0.98 | 0.99 | 0.98 |
| TtgR | 0.93 | 0.91 | 0.91 |
| MphR | 0.97 | 0.97 | 0.97 |
| RolR | 0.95 | 0.92 | 0.93 |
