# Supplementary material for: Deep mutational scanning and machine learning reveal structural and molecular rules governing allosteric hotspots in homologous proteins
Source: eLife. 2022 Oct 13;11:e79932. doi: 10.7554/eLife.79932 (PMC9662819; doi:10.7554/eLife.79932)
Supplement: Supplementary file 3. [file elife-79932-supp3.docx]

| **Cluster** | **TetR** | | | **TtgR** | | | **MphR** | | | **RolR** | | |
| --- | --- | --- | --- | --- | --- | --- | --- | --- | --- | --- | --- | --- |
|  | Residues | Hotspots | Percent | Residues | Hotspots | Percent | Residues | Hotspots | Percent | Residues | Hotspots | Percent |
| 1 | 36 | 0 | 0.0 | 27 | 2 | 7.4 | 24 | 3 | 12.5 | 28 | 5 | 17.9 |
| 2 | 55 | 16 | 29.1 | 51 | 22 | 43.1 | 47 | 17 | 36.2 | 46 | 18 | 39.1 |
| 3 | 41 | 14 | 34.1 | 28 | 6 | 21.4 | 23 | 6 | 26.1 | 35 | 10 | 28.6 |
| 4 | 37 | 13 | 35.1 | 29 | 10 | 34.5 | 36 | 10 | 27.8 | 45 | 17 | 37.8 |
| 5 | 26 | 10 | 38.5 | 9 | 2 | 22.2 | 27 | 3 | 11.1 | 28 | 4 | 14.3 |
| 6 | 27 | 0 | 0.0 | 37 | 10 | 27.0 | 45 | 15 | 33.3 | 31 | 12 | 38.7 |
| 7 | 43 | 8 | 18.6 | 57 | 20 | 35.1 | 22 | 8 | 36.4 | 36 | 15 | 41.7 |
| 8 | 41 | 18 | 43.9 | 32 | 11 | 34.4 | 35 | 11 | 31.4 | 26 | 8 | 30.8 |
| 9 | 20 | 10 | 50.0 | 16 | 0 | 0.0 | 14 | 12 | 85.7 | 28 | 9 | 32.1 |
| 10 | 20 | 14 | 70.0 | 53 | 14 | 26.4 | 44 | 24 | 54.5 | 60 | 26 | 43.3 |

Supplementary File 3: Cluster Rankings
