# Supplementary material for: Deep mutational scanning and machine learning reveal structural and molecular rules governing allosteric hotspots in homologous proteins
Source: eLife. 2022 Oct 13;11:e79932. doi: 10.7554/eLife.79932 (PMC9662819; doi:10.7554/eLife.79932)
Supplement: Supplementary file 4. [file elife-79932-supp4.docx]

Supplementary File 4. Template information

|  | Template (PDB code) | Sequence identity (%) | RMSD (Å) |
| --- | --- | --- | --- |
| TetR | 1A6I | 68.8 | 1.52 |
|  | 3BQY | 29.8 | 3.07 |
| MphR | 6U18 | 97.9 | 0.91 |
|  | 4GFK | 15.4 | 3.52 |
| RolR | 3AQS | 98.9 | 0.93 |
|  | 2HYT | 22.4 | 3.37 |
| TtgR | 3LHQ | 36.9 | 2.26 |
|  | 2NX4 | 17.7 | 3.44 |
