# Supplementary figures and images for: Deep mutational scanning and machine learning reveal structural and molecular rules governing allosteric hotspots in homologous proteins
Source: eLife. 2022 Oct 13;11:e79932. doi: 10.7554/eLife.79932 (PMC9662819; doi:10.7554/eLife.79932)

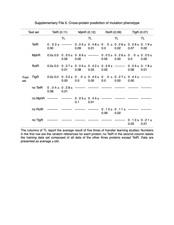

Supplement: Supplementary file 5. [file elife-79932-supp5.zip › Supplementary File 5.pages_FILES/preview-web.jpg]

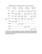

Supplement: Supplementary file 5. [file elife-79932-supp5.zip › Supplementary File 5.pages_FILES/preview-micro.jpg]

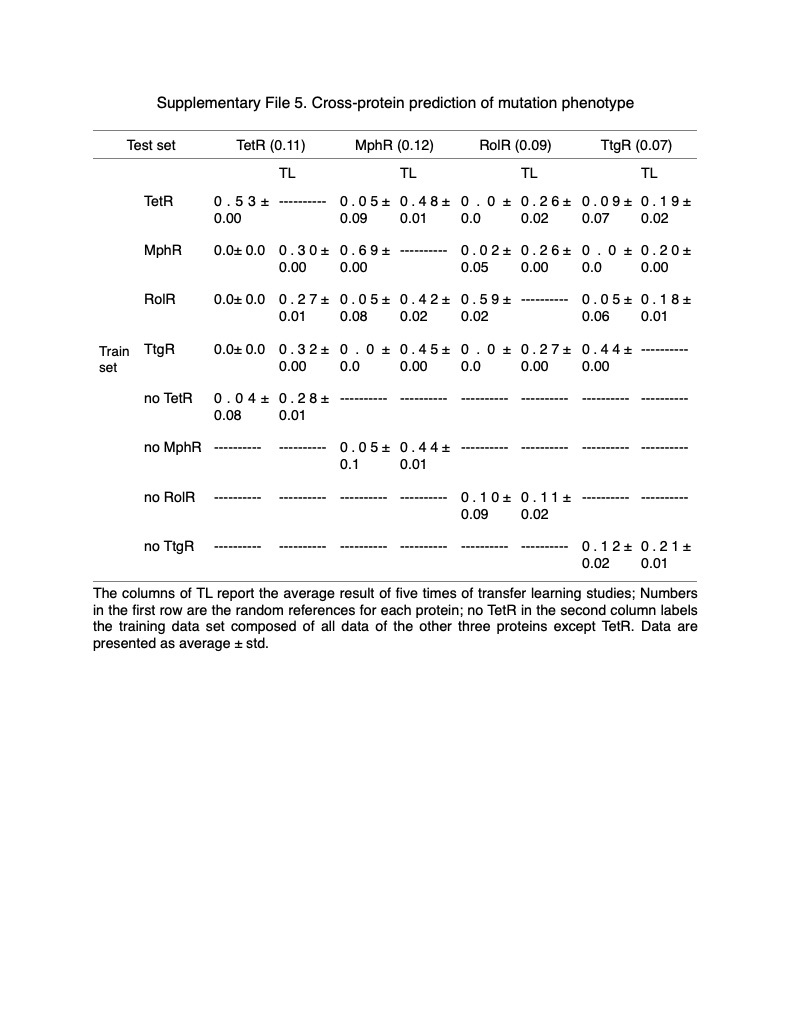

Supplement: Supplementary file 5. [file elife-79932-supp5.zip › Supplementary File 5.pages_FILES/preview.jpg]

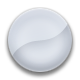

Supplement: Supplementary file 5. [file elife-79932-supp5.zip › Supplementary File 5.pages_FILES/Data/bullet_gbutton_gray-30.png]

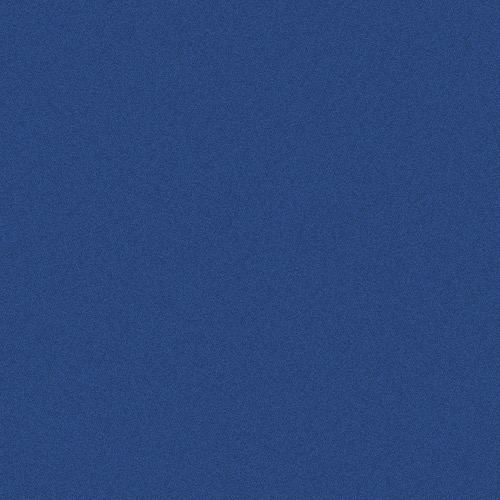

Supplement: Supplementary file 5. [file elife-79932-supp5.zip › Supplementary File 5.pages_FILES/Data/PresetImageFill0-24.jpg]

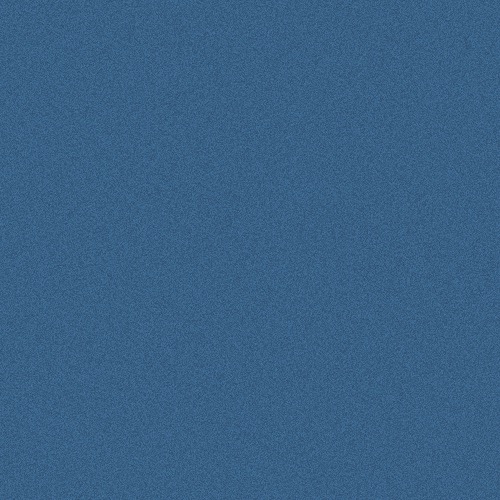

Supplement: Supplementary file 5. [file elife-79932-supp5.zip › Supplementary File 5.pages_FILES/Data/PresetImageFill4-28.jpg]

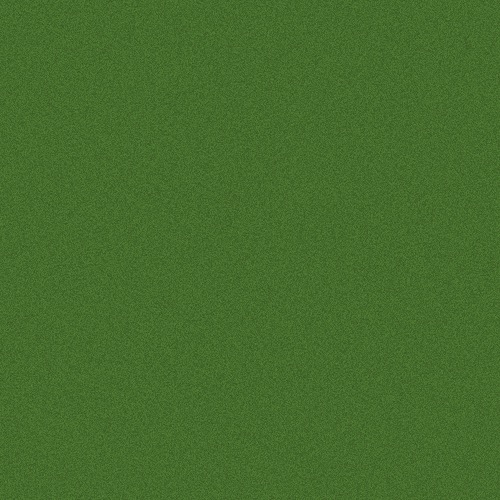

Supplement: Supplementary file 5. [file elife-79932-supp5.zip › Supplementary File 5.pages_FILES/Data/PresetImageFill5-29.jpg]

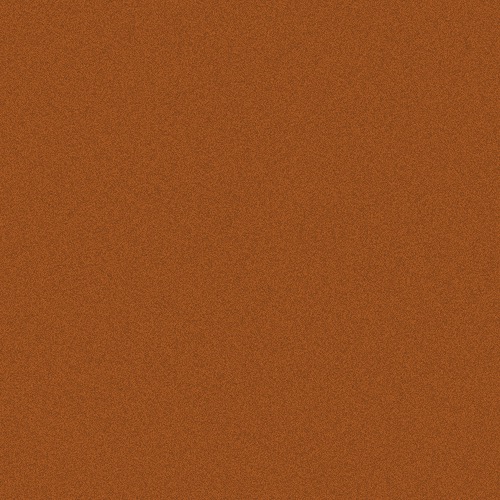

Supplement: Supplementary file 5. [file elife-79932-supp5.zip › Supplementary File 5.pages_FILES/Data/PresetImageFill1-25.jpg]

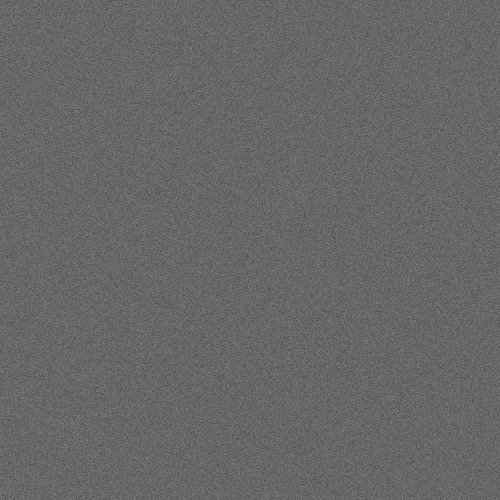

Supplement: Supplementary file 5. [file elife-79932-supp5.zip › Supplementary File 5.pages_FILES/Data/PresetImageFill2-26.jpg]

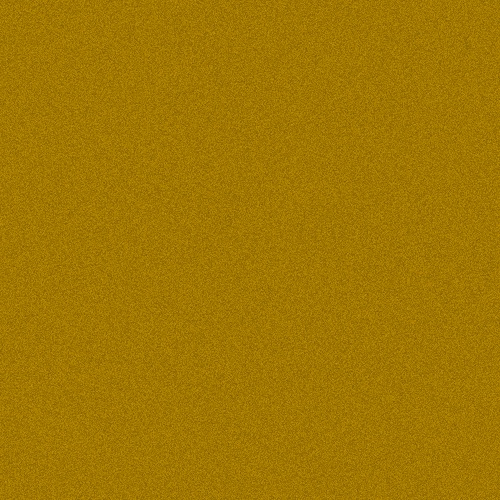

Supplement: Supplementary file 5. [file elife-79932-supp5.zip › Supplementary File 5.pages_FILES/Data/PresetImageFill3-27.jpg]
